# α-Lipoic Acid Inhibits IL-8 Expression by Activating Nrf2 Signaling in *Helicobacter pylori*-infected Gastric Epithelial Cells

**DOI:** 10.3390/nu11102524

**Published:** 2019-10-19

**Authors:** Seoyeon Kyung, Joo Weon Lim, Hyeyoung Kim

**Affiliations:** Department of Food and Nutrition, Brain Korea 21 PLUS Project, College of Human Ecology, Yonsei University, Seoul 03722, Korea; loveminb@naver.com (S.K.); jwlim11@yonsei.ac.kr (J.W.L.)

**Keywords:** α-lipoic acid, *Helicobacter pylori*, interleukine-8, nuclear factor erythroid-2-related factor 2, heme oxygenase-1

## Abstract

*Helicobacter pylori (H. pylori)* causes gastritis and gastric cancers. Oxidative stress is involved in the pathological mechanism of *H. pylori*-induced gastritis and gastric cancer induction. Therefore, reducing oxidative stress may be beneficial for preventing the development of *H. pylori*-associated gastric diseases. Nuclear factor erythroid-2-related factor 2 (Nrf2) is a crucial regulator for the expression of antioxidant enzyme heme oxygenase-1 (HO-1), which protects cells from oxidative injury. α-Lipoic acid (α-LA), a naturally occurring dithiol, shows antioxidant and anti-inflammatory effects in various cells. In the present study, we examined the mechanism by which α-LA activates the Nrf2/HO-1 pathway, suppresses the production of pro-inflammatory cytokine interleukine-8 (IL-8), and reduces reactive oxygen species (ROS) in *H. pylori*-infected AGS cells. α-LA increased the level of phosphorylated and nuclear-translocated Nrf2 by decreasing the amount of Nrf2 sequestered in the cytoplasm by complex formation with Kelch-like ECH1-associated protein 1 (KEAP 1). By using exogenous inhibitors targeting Nrf2 and HO-1, we showed that up-regulation of activated Nrf2 and of HO-1 results in the α-LA-induced suppression of interleukin 8 (IL-8) and ROS. Consumption of α-LA-rich foods may prevent the development of *H. pylori*-associated gastric diseases by decreasing ROS-mediated IL-8 expression in gastric epithelial cells.

## 1. Introduction

α-Lipoic acid (α-LA) is an endogenous dithiol found in small quantities in all foods but is slightly enriched in spinach, broccoli and some meats [1]. α-LA is widely used as a dietary supplement because it is regarded as an ideal antioxidant agent owing to its ability to scavenge free radicals, chelate transition metal ions, and up-regulate the expression of antioxidant genes [2]. α-LA has been shown to confer a protective effect against various diseases, including Alzheimer’s disease and diabetes [3,4,5,6]. Oral administration of α-LA has modulated insulin sensitivity in patients with type-2 diabetes mellitus [3,4]. α-LA treatment has improved cognitive functioning in patients with Alzheimer's disease [5,6]. Thus, determination of the action mechanism(s) of α-LA is of special importance.

In previous work focusing on the effect of α-LA on *Helicobacter pylori (H. pylori)* infection-induced oxidative stress in gastric epithelial cells, we showed that α-LA decreases ROS levels and suppresses NADPH oxidase activity and the inflammatory signaling pathways mediated by mitogen-activated protein kinases, janus kinase/signal transducers and activators of transcription (JAK/STAT) and nuclear factor kappa light chain enhancer of activated B cells (NF-kB) [7,8]. In the present study, we have focused on the effect of α-LA on nuclear factor erythroid 2-related factor 2 (Nrf2), which is a crucial transcriptional regulator of antioxidant enzymes such as heme oxygenase-1 (HO-1), thioredoxins and peroxiredoxins (Prxs) [9]. 

Nrf2 is retained in the cytoplasm at low levels by the redox-sensor Kelch-like ECH-associated protein 1 (KEAP 1), which in turn facilitates Nrf2 ubiquitination and subsequent degradation [10]. Oxidative stress results in release of Nrf2 from KEAP 1, which allows Nrf2 to translocate to the nucleus where it binds to antioxidant response elements (AREs) [11]. AREs are sites within promoter regions of genes that bind transcriptional factors such as Nrf2 and thereby facilitate the expression of genes encoding phase II detoxification enzymes and anti-oxidant enzymes such as HO-1 [12]. High levels of ROS-scavenging enzymes were frequently found in cancer cells. Mutant KEAP 1 is present in non-small-cell lung cancer (NSCLC) cell lines and in NSCLC patients, which leads to constitutive activation of Nrf2 function and cytoprotection against ROS-generating radiotherapy and chemotherapeutic agents [13]. 

The anti-oxidant effect of HO-1 has been demonstrated for a variety cell types. For example, the proton pump-inhibitor, lansoprazole, exerts its anti-inflammatory effect in gastric mucosal cells by inducing HO-1 expression via Nrf2 activation and KEAP 1 oxidation [14]. The antioxidant curcumin exerts its antioxidant and anti-inflammatory effects in vascular epithelial cells by up-regulating HO-1 expression [15]. Likewise, the anti-oxidant α-LA protects monocytes [16] and retinal neuronal cells [17] from oxidative stress by up-regulating HO-1 expression. 

*H. pylori* is a Gram-negative bacterium, usually acquired during childhood, whose natural habitat is the gastric lumen. *H. pylori* is accepted as the most important cause of gastritis and peptic ulcer in humans [18]. Furthermore, its important role in the pathogenesis of gastric cancer as well as in several extra-gastroduodenal diseases has been confirmed [19,20]. Oxidative stress is an important component of *H. pylori*-induced chronic infection [21]. This bacterium colonizes human gastric mucosa by infiltration of neutrophils into the epithelial cell layer. This process is dependent on the production of pro-inflammatory cytokines, especially the chemotactic cytokine interleukine-8 (IL-8). The expression of IL-8 varies depending on the density or strain of *H. pylori* that infects the host cells [22]. IL-8 acts as a powerful mediator of the inflammatory response by activating and attracting neutrophils, basophils and T cells to the site of infection [23,24]. This generates high levels of ROS at the site [25], which in turn causes oxidative stress-induced gastric damage [26]. Several studies have demonstrated that the ROS produced by *H. pylori* mediates the expression of IL-8 [27,28,29]. Therefore, therapeutic agents that inhibit ROS production or that scavenge ROS could serve in the treatment of *H. pylori*-associated gastric mucosal inflammation.

The purpose of the present study is to determine the mechanism(s) by which α-LA inhibits ROS-mediated expression of pro-inflammatory cytokine IL-8 in *H. pylori*-infected gastric epithelial cells. Herein, we report experimental evidence for α-LA-induced reduction in ROS and IL-8 and up-regulation of HO-1 via Nrf2 activation in *H. pylori*-infected gastric epithelial AGS cells.

## 2. Materials and Methods 

### 2.1. Reagents 

α-LA (R- α-LA) and a Nrf2 inhibitor, trigonelline, were purchased from Sigma-Aldrich (St. Louis, MO, USA) and were dissolved in 0.5 M ethanol and distilled water, respectively. A HO-1 inhibitor protoporphyrin (ZnPP) was purchased from Santa Cruz Biotechnology (sc-2000329, Santa Cruz Biotechnology, Santa Cruz, CA, USA) and was dissolved in dimethyl sulfoxide (DMSO). 

### 2.2. Cell Line and Culture Conditions 

Gastric epithelial AGS cells (gastric adenocarcinoma, ATCC CRL 1739, Rockvile, MD, USA) were purchased from the American Type Culture Collection (Rockvile, MD, USA). The cells were grown in RPMI 1640 medium (GIBCO, Grand Island, NY, USA) supplemented with 10% fetal bovine serum, 2 mM glutamine, 100 U/mL penicillin, and 100 μg/mL streptomycin (Sigma-Aldrich, St. Louis, MO, USA). The cells were grown under a humidified atmosphere of 95% air and 5% CO_2_ at 37 °C.

### 2.3. Cell Culture with H. pylori Infection 

*H. pylori*, strain NCTC 11637, was obtained from the American Type Culture Collection. The bacterium was grown under microaerophilic conditions at 37 °C, using an anaerobic chamber (BBL Campy Pouch System, Becton Dickinson Microbiology Systems, Franklin Lakes, NJ, USA).

AGS cells were seeded and cultured overnight to reach 80% confluency. Prior to *H. pylori* infection, the cells were washed once with culture medium containing no antibiotics. Whole *H. pylori* was suspended in antibiotic-free RPMI 1640 medium supplemented with 10% fetal bovine serum, and then treated to the AGS cells. The ratio of AGS cell: *H. pylori* was 1:100. The ratio of AGS cell: *H. pylori,* 1:100, was adapted from our previous study showing high expression of IL-8 in gastric epithelial AGS cells infected with *H. pylori*, NCTC11637 [30].

### 2.4. Experimental Protocol

To investigate the effect of α-LA on Nrf2 activation, dissociation of KEAP1-Nrf2 complex, nuclear translocation of Nrf2, and HO-1 expression, the AGS cells (5 × 10^4^/mL) were treated with a solution of α-LA in 0.5 M ethanol to produce a final α-LA concentration of 5 μM and cultured for 2 h (for the determination of the KEAP 1-Nrf2 complex and nuclear translocation of Nrf2) and for 8 h (for determination of KEAP 1, HO-1 and phosphorylated and total Nrf2 in whole-cell extracts or nuclear extracts). 

To determine the effect of α-LA on the expression of IL-8 and HO-1 and ROS levels, AGS cells were treated with α-LA (at final concentrations of 2.5 and 5 μM) for 2 h and incubated with *H. pylori* for 1 h (for the determination of HO-1 and ROS), 12 h (for IL-8 mRNA), and 24 h (for IL-8).

To assess the involvement of Nrf2 and HO-1 in the inhibitory effect of α-LA on *H. pylori*-induced IL-8 expression, the cells were treated with trigonelline (1 μM) or ZnPP (2 μM) in the presence of α-LA (5 μM) and incubated with *H. pylori* for 1 h (for the determination of HO-1 and ROS), 12 h (for IL-8 mRNA), and 24 h (for IL-8). For each experiment, the amount of a vehicle was less than 0.5%. A control, in which the vehicle alone was added, was carried out in parallel. 

### 2.5. Measurement of Intracellular ROS Levels

The cells were treated with 10 μg/mL of dichlorofluorescein diacetate (DCF-DA; Sigma-Aldrich) and incubated for 30 min under an atmosphere of 5% CO_2_/95% air at 37 °C. The intensities of DCF fluorescence at 535 nm (excitation at 495 nm) were measured with a Victor 5 multi-label counter (PerkinElmer Life and Analytical Sciences, Boston, MA, USA). The intracellular ROS levels were normalized to cell numbers and expressed as the relative increase.

### 2.6. Real-time PCR Analysis 

Cellular RNA was isolated by using the reagent TRI (Molecular Research Center, Inc., Cincinnati, OH, USA). The RNA was converted to cDNA by reverse transcription using a random hexamer as template, MuLV reverse transcriptase (Promega, Madison, WI, USA) as catalyst and the reaction conditions: 23 °C for 10 min, 37 °C for 60 min and 95 °C for 5 min. The cDNA was used for real-time PCR. The IL-8 primers 5’-ATGACTTCCAAGCTGGCCGTGGCT-3’ (forward primer) and 5’-TCTCAGCCCTCTTCAAAAACTTCT-3’ (reverse primer) were used to generate a 297 bp PCR product. For β-actin, the forward primer used is 5’-ACCAACTGGGACGACATGGAG-3’ and the reverse primer used is 5’-GTGAGGATCTTCATGAGGTAGTC-3’, giving a 349 bp PCR product. cDNA was amplified by 45 repeat denaturation cycles at 95 °C for 30 s, annealing at 55 °C for 30 s, and extension at 72 °C for 30 s. During the first cycle, the 95 °C step was extended to 3 min. The β-actin gene was amplified in the same manner to serve as the reference gene.

### 2.7. Enzyme-Linked Immunosorbent Assay (ELISA)

The AGS cells (1.5 × 10^5^ / well) were seeded in 6-well plates. The culture medium was centrifuged at 15,000 rpm for 15 min at 4 °C. The supernatant was collected for quantitating IL-8 with an enzyme-linked immunosorbent assay (ELISA) kit (Invitrogen Corporation, Carlsbad, CA, USA) according to the manufacturer’s instructions.

### 2.8. Preparation of Whole-Cell Extracts and Nuclear Extracts

Cells were harvested into phosphate buffered saline (PBS) and pelleted by centrifugation at 5000× *g* for 15 min. The cell pellets were re-suspended in lysis buffer containing 10 mM Tris (pH 7.4), 1% NP-40 and a commercial protease inhibitor complex (Complete; Roche, Mannheim, Germany). Cells were lysed through a 1 mL syringe using several rapid strokes. The lysates were centrifuged at 13,000× *g* for 15 min. The supernatants were used as whole-cell extracts. To prepare the nuclear extracts, the cell pellets were re-suspended with 30 μL of hypotonic buffer containing 10 mM HEPES (pH 7.9), 1.5 mM MgCl_2_, 10 mM KCl, 0.5 mM DTT, 0.5 mM PMSF, and 0.2% NP-40. The extracts were centrifuged at 13,000× *g* and 4 °C for 20 min. The pellets were re-suspended in 30 μL of extraction buffer containing 20 mM HEPES (pH 7.9), 420 mM NaCl, 0.2 mM EDTA, 1.5 mM MgCl_2_, 25% glycerol, 0.5 mM DTT, and 0.5 mM PMSF. After centrifugation at 13,000× *g* and 4 °C for 20 min, the supernatants were used as the nuclear extracts. Bradford assay (Bio-Rad Laboratories, Hercules, CA, USA) was used for determination of protein.

### 2.9. Western Blot Analysis 

Whole-cell extracts (6–40 μg/per lane) were loaded onto 8–10% SDS polyacrylamide gels and separated by electrophoresis under reducing conditions. The proteins were then transferred by electroblotting and verified by using reversible staining with Ponceau S. The membranes were blocked using 3% non-fat dry milk in TBS-T (Tris-buffered saline and 0.2% Tween 20). The proteins were detected with antibodies for Nrf2 (sc-722, Santa Cruz Biotechnology, Dallas, TX, USA), p-Nrf2 (ab76026, Abcam, Cambridge, UK), KEAP 1 (sc-365626, Santa Cruz Biotechnology), and HO-1 (ADI-SPA-895, Enzo Life Science Inc., Farmingdale, NY, USA). The antibodies were diluted in TBS-T containing 3% dry milk and incubated with the membrane overnight at 4 °C. After washing with TBS-T, primary antibodies were detected with horseradish peroxidase-conjugated secondary antibodies (anti-rabbit, anti-mouse, anti-goat) and visualized by using the enhanced chemiluminescence detection system (Santa Cruz Biotechnology, Dallas, TX, USA) and BioMax MR film (Kodak, Rochester, NY, USA). The protein levels were compared to that of the loading control actin, or histone H1.

### 2.10. Immunoprecipitation of the Nrf2-KEAP 1 Complex

α-LA (5 μM)-treated and untreated AGS cells were lysed in 500 μM of immunoprecipitation buffer containing 10 mM Tris-HCl (pH 7.4), 100 mM NaCl, 1 mM EDTA, 1 mM EGTA, Complete, 0.5% NP-40, 0.5% sodium deoxycholate, and 10% glycerol. Then, cells were centrifuged at 15,000× *g* for 15 min. Polylconal antibody and protein G-agarose were added to the cleared supernatant, and the mixture was incubated overnight at 4 °C. The protein G-antibody-antigen complex was then collected by washing four times with immunoprecipitation buffer containing 150 mM NaCl, 10 mM Tris-HCl (pH 7.4), 1 mM EDTA, 1 mM EGTA, 0.5 % NP-40, and 0.5 % sodium deoxycholate. The pellet was resuspended in 50 μL of SDS sample buffer and boiled for 10 min. The preparation was then subjected to Western blot analysis.

### 2.11. Immunofluorescence Staining

The cells were treated with α-LA for 2 h on slide glasses and then fixed with cold 100% methanol. The fixed cells were permeabilized with 0.1% Triton X-100 in PBS for 5 min, blocked with 0.1% gelatin and 1% bovine serum albumin in PBS for 1 h, and then incubated for 1 h with the primary antibody for Nrf2. After washing with PBS, the cells were incubated with rhodamine-conjugated mouse anti-rabbit IgG antibody (sc-2492, Santa Cruz Biotechnology) for 1 h. After removal of the secondary antibody, the cells were washed with PBS and covered with the antifade medium Vectashield containing 4’,6-diamidino-2-phenylindole (DAPI). The preparations were stored for 30 min to allow saturation with DAPI. The cells stained with Rhodamine-conjugated antibody were examined with a laser scanning confocal microscope (Zeiss LSM 880, Carl Zeiss Inc., Thornwood, NY, USA) and photographed.

### 2.12. Statistical Analysis

One-way ANOVA and Newman-Keul’s post-hoc test were used. All results are expressed as the mean ± S.E. of four different experiments. A *p*-value of 0.05 or less was considered to be statistically significant.

## 3. Results

### 3.1. α-LA Increases Expression, Phosphorylation, and Nuclear Translocation of Nrf2, and Expression of HO-1 in AGS Cells

**Figure 1:**
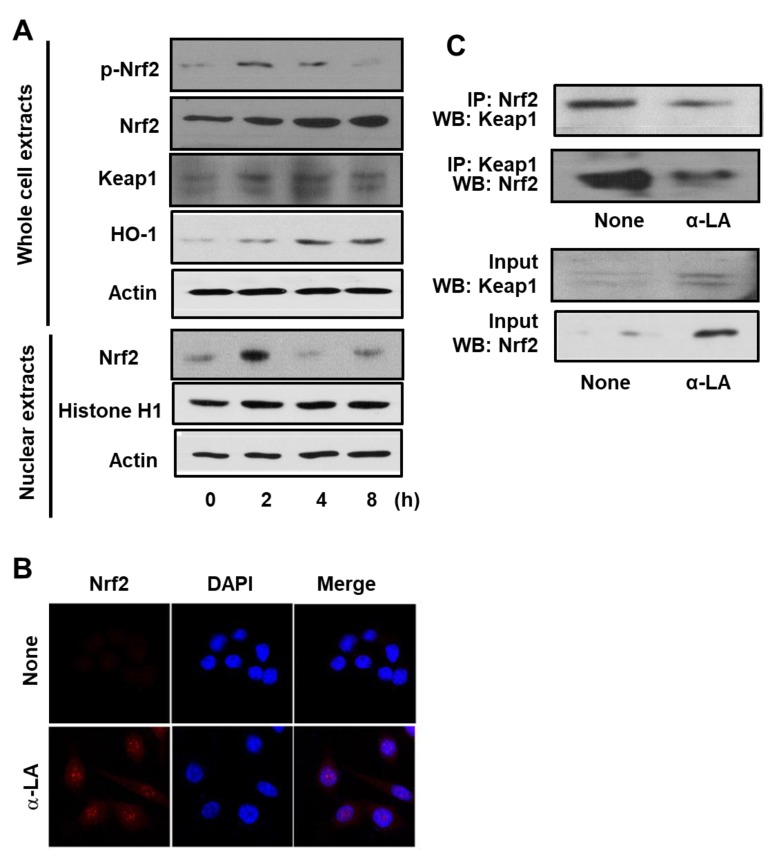
Determination of the impact of α-LA on the levels of activated Nrf2, KEAP 1-bound Nrf2, KEAP 1, and HO-1 in AGS cells. (**A**) Western blots of whole-cell extracts (upper panel) or nuclear extracts (bottom panel) with actin serving as the loading control and histone H1 serving as the index for the nuclear extracts. The cells were treated with 5 μM α-LA for the indicated time periods. (**B**) Confocal microscope images of AGS cells treated with 5 μM α-LA for 2 h followed by immunofluorescence staining of the fixed cells. Nrf2 was visualized using fluorescein rhodamin-conjugated anti-rabbit IgG antibody (red) with DAPI counter staining (blue) of the same field. “None” refers to the cells treated with the vehicle for α-LA (0.5 M ethanol) alone. (**C**) Western blots of whole-cell extracts (lower panel) and whole-cell extract-derived immunoprecipitates obtained using the anti-Nrf2 and anti-KEAP 1 antibodies for precipitation (IP) and visualization (WB; western blot analysis) as indicated (upper panel). The cells were treated with 5 μM α-LA for 2 h. Input is used as the control for protein expression. “None” refers to the cells treated with the vehicle for α-LA (0.5 M ethanol) only.

Prior to determining the effect of α-LA on *H. pylori*-infected cells, we measured the effect of α-LA on uninfected cells. Firstly, we measured α-LA-induced changes in Nrf2 expression, phosphorylation, and nuclear translocation. Accordingly, Western blot analysis of whole-cell extracts and of nuclear extracts was carried out using AGS cells treated with α-LA for 2, 4 and 8 h. Figure 1A shows that the level of Nrf2 in the whole-cell extracts increased in a time-dependent manner over the 8 h period. In contrast, the level of p-Nrf2 in the whole-cell extracts, and Nrf2 in the nuclear extracts, reached a maximum at 2 h and then declined in a time-dependent manner (Figure 1A). In order to verify the impact of α-LA on Nrf2 nuclear translocation, AGS cells were incubated with α-LA for 2 h and then subjected to immunofluorescence staining. Whereas the level of the nuclear marker DAPI was not changed by α-LA, the level of Nrf2 in the nucleus was increased by α-LA (Figure 1B). 

Next, the impact of α-LA on the expression KEAP 1 and HO-1 was examined by Western blot analysis of whole-cell extracts. As shown in Figure 1A, the levels of KEAP 1 and HO-1 were increased in a time-dependent manner over the 8 h period. These results indicated that α-LA activates Nrf2, and that this activation up-regulates HO-1 expression. Because KEAP 1 sequesters Nrf2 in the cytoplasm, we carried an additional experiment (see below) to determine if α-LA alters the level of the Nrf2-KEAP 1 complex.

### 3.2. α-LA Decreases Interaction between KEAP1 and Nrf2 in AGS Cells

The impact of α-LA on the level of Nrf2-KEAP 1 association in AGS cells was examined by carrying out immunoprecipitation (IP) of the complex with anti-KEAP 1 antibody or anti-Nrf2 antibody followed by Western blot analysis (WB) using anti-Nrf2 antibody and anti-KEAP 1 antibody, respectively. The results obtained for cells treated with α-LA for 2 h are reported in Figure 1C, along with the results obtained for the control experiment (“None”) in which α-LA was excluded. Whereas the treatment of cells with α-LA increased the level of KEAP 1 and Nrf2 present in whole-cell extracts (Figure 1C, lower panel), it decreased the level of KEAP 1 and Nrf2 present in the immunoprecipitate (Figure 1C, upper panel). This finding indicates that α-LA can increase Nrf2 activity by inhibiting KEAP 1-mediated sequestration of Nrf2.

### 3.3. α-LA Decreases IL-8 Expression and ROS Levels, but Increases HO-1 Expression in H. pylori-infected AGS Cells

**Figure 2:**
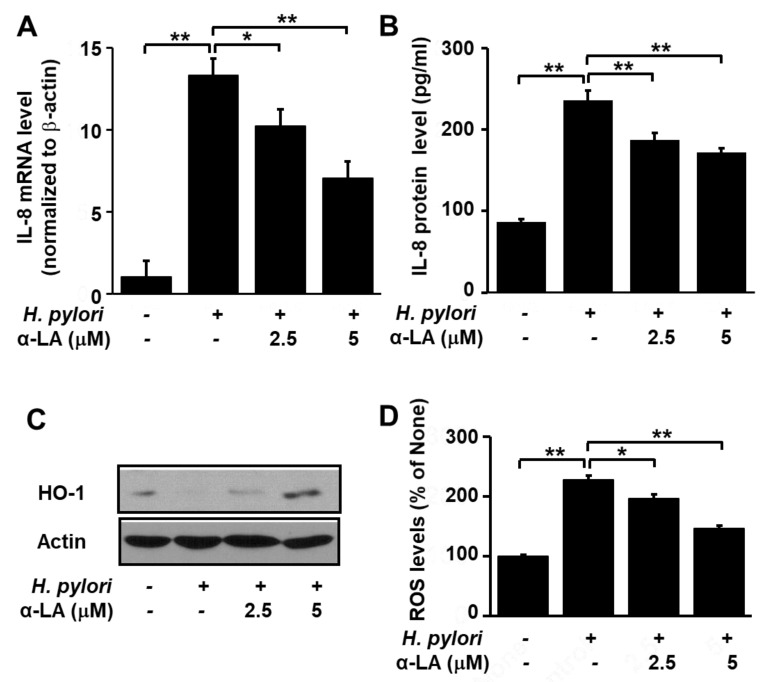
Determination of the impact of α-LA on the levels of HO-1, IL-8 and ROS in *H. pylori*-infected AGS cells. The cells were pretreated with the indicated concentration of α-LA for 8 h, before infection with *H. pylori*. (**A**) The amount of IL-8 mRNA determined by real-time PCR analysis and normalized to actin mRNA. (**B**) The quantity of IL-8 in the cell culture medium determined by ELISA. Data are expressed as the mean ± S.E. of four different experiments. (**C**) Western blot of whole-cell extracts using β-actin as the loading control. (**D**) ROS levels determined by fluorescent DCF. Intracellular ROS is expressed as the relative increase. The value for cells without *H. pylori* infection in the absence of α-LA treatment is set as 100%. * *p* < 0.05, ** *p* < 0.01.

As shown in Figure 2, *H. pylori* infection increased IL-8 gene expression at the mRNA (Figure 2A) and protein (Figure 2B) levels. However, pretreatment of the cells with α-LA significantly reduced these increases in a dose-dependent manner. In addition, whereas *H. pylori* infection lowered the HO-1 level, treatment of the cells with α-LA prior to infection resulted in a net increase in the HO-1 level (Figure 2C). 

Next, we compared the levels of ROS in *H. pylori*-infected AGS cells pretreated with α-LA, using α-LA-untreated and *H. pylori*-infected cells as the control. Figure 2D shows that α-LA reduced, in a dose dependent manner, the up-regulation of ROS in *H. pylori*-infected AGS cells. Together, these findings suggest that α-LA reduces oxidative stress in *H. pylori*-infected cells by reducing ROS and IL-8 and increasing HO-1 in AGS cells. 

### 3.4. Nrf2 Inhibitor Trigonelline Abolishes the Effect of α-LA on IL-8, HO-1 and ROS Levels in H. pylori-infected AGS Cells 

**Figure 3:**
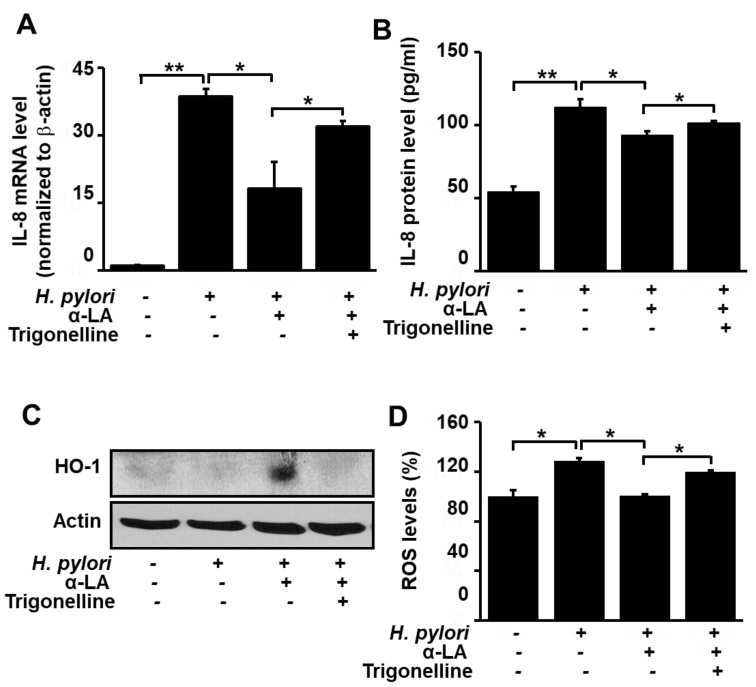
Determination of the impact of the Nrf2 inhibitor trigonelline on IL-8 gene expression and ROS levels in *H. pylori*-infected AGS cells treated with α-LA. The cells were treated with 5 μM α-LA and 1 μM trigonelline for 8 h, and then infected with *H. pylori*. (**A**) The amount of IL-8 mRNA determined by real-time PCR analysis and normalized to cellular actin mRNA. (**B**) The amount of IL-8 in the culture medium determined by ELISA. Data are expressed as the mean ± S.E. of three different experiments. (**C**) Western blot analysis for whole-cell extracts developed with anti-OH antibody. Actin was used as a loading control. (**D**) Cellular ROS levels determined by measuring the level of fluorescent DCF. The level of intracellular ROS is expressed as the relative increase. The value for cells without *H. pylori* infection in the absence of α-LA is set as 100%. * *p* < 0.05, ** *p* < 0.01.

Having shown that α-LA reduces the levels of ROS and IL-8, and increases the level of HO-1, in *H. pylori*-infected AGS cells, we next turned our attention to determining if these effects are the result of the α-LA-induced activation of Nrf2 observed with the uninfected cells (Figure 1). For this purpose, the Nrf2 inhibitor trigonelline was used to block the effect of α-LA-induced Nrf2 activation. Figure 3A and 3B show that in the presence of the inhibitor, the capacity for α-LA to diminish the *H. pylori*-induced increase in IL-8 gene expression at the mRNA level (Figure 3A) and protein level (Figure 3B) was reduced. Likewise, trigonelline diminished the α-LA-induced increase in HO-1 expression (Figure 3C), as well as the α-LA-induced decrease in ROS (Figure 3D). These findings support the role of Nrf2 in mediating α-LA-induced down-regulation of IL-8 and ROS and up-regulation of HO-1 in *H. pylori*-infected AGS cells. 

### 3.5. HO-1 Inhibitor ZnPP Inhibits the Effect of α-LA on ROS Levels and Expression of IL-8 in H. pylori-infected AGS Cells 

**Figure 4:**
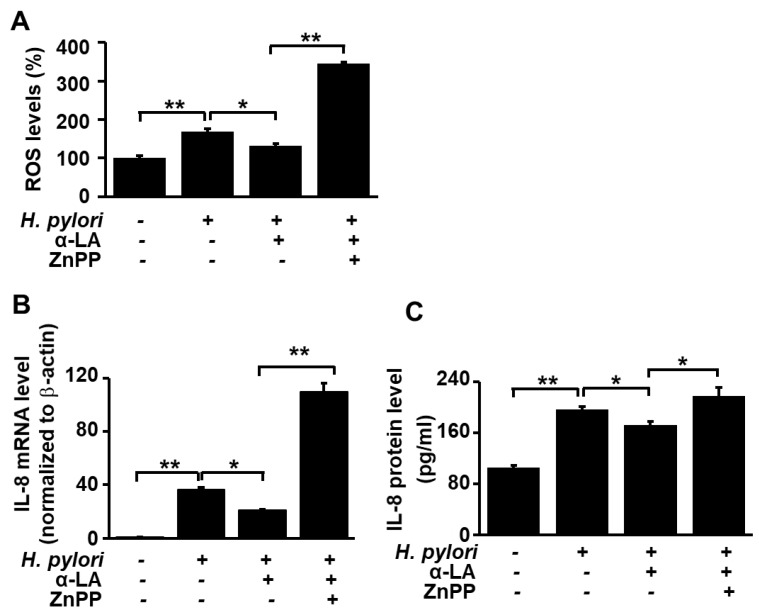
Determination of the impact of HO-1 inhibitor ZnPP on IL-8 gene expression and ROS levels in *H. pylori*-infected AGS cells treated with α-LA. The cells were co-treated with 5 μM α-LA and 2 μM ZnPP for 8 h and then infected with *H. pylori*. (**A**) Cellular ROS levels determined by measuring the level of fluorescent DCF. The intracellular ROS is expressed as the relative increase. The value for cells without *H. pylori* infection in the absence of α-LA was set as 100%. (**B**) IL-8 mRNA levels determined by real-time PCR analysis and normalized to actin mRNA. (**C**) The level of IL-8 in cell culture medium determined by ELISA. Data are expressed as the mean ± S.E. of three different experiments. * *p* < 0.05, ** *p* < 0.01.

Having shown that α-LA increases the level of HO-1 in AGS cells in which HO-1 levels were reduced as a result of the *H. pylori* infection, we carried out experiments to test whether the α-LA-induced decrease in ROS and IL-8 resulting from *H. pylori* infection is the result of the α-LA-induced increase in HO-1. Accordingly, infected AGS cells were treated with α-LA in the presence and absence of the HO-1 inhibitor ZnPP. Figure 4 shows that ZnPP suppressed the α-LA-induced decrease in ROS levels (Figure 4A) and decrease in IL-8 expression at both the mRNA (Figure 4B) and protein (Figure 4C) levels. These results demonstrate that α-LA inhibits IL-8 gene expression and reduces the ROS levels via up-regulation of HO-1 expression in *H. pylori*-infected cells. 

## 4. Discussion

In the present study, we found that α-LA activates the Nrf2/HO-1 pathway by decreasing the interaction between Nrf2 and KEAP 1. We also discovered that the levels of the pro-inflammatory agents ROS and IL-8 are reduced by α-LA as a result of its activation the Nrf2/HO-1 pathway. These findings are consistent with the results obtained from previous studies carried out with other disease models. In particular, a recent study demonstrated that α-LA improved neurobehavioral function by up-regulation of Nrf2 expression and its downstream protein factors including HO-1 and quinine oxidoreductase-1 (NQO-1) after traumatic brain injury in rats [31]. In addition, α-LA treatment decreased cell death and ROS production in urban particulate matter (UPM)-exposed fibroblasts via the Nrf2, HO-1, and NQO-1 pathways. Also, α-LA treatment abrogated increases in IL-6 and IL-8 levels induced by UPM in nasal fibroblasts [32]. Moreover, it has been reported that α-LA ameliorated hepatic steatosis in rats of diabetes fed with high fat by increasing antioxidant defense systems through Nrf2 and consequently decreasing oxidative stress and hepatic TNF-α [33]. Nab-paclitaxel is an anti-cancer drug, but its side effect, peripheral neuropathy, affects both the quality of life and the survival of cancer patients. Interestingly, *α*-LA could prevent oxidative stress and peripheral neuropathy in Nab-paclitaxel-treated rats through the Nrf2 signaling pathway without diminishing chemotherapeutic effect [34]. In the studies on Nrf2 and KEAP 1, the Nrf2 activators sequestosome-1, 5,6-dihydrocyclopenta-1,2-dithiole-3-thione and sulforaphane activate Nrf2 by decreasing the interaction between Nrf2 and KEAP 1 [35,36]. 

Previous studies carried out with macrophages, dendritic cells, and gastric cancer cells have indicated that *H. pylori* infection up-regulates HO-1 expression, thereby providing a protective effect against increased levels of ROS [36,37,38]. In the present study, we observed that *H. pylori* infection of the gastric epithelial AGS cells results in HO-1 down-regulation, in agreement with the findings reported by Gobert et al. [39] The down-regulation of the Nrf2/HO-1 pathway observed with the AGS cell line can be attributed to the action of the heat-shock protein B (HspB) present in certain strains of *H. pylori* [40], including the strain NCTC11637 [40] used in our studies. Specifically, AGS cells transfected with the *H. pylori* HspB gene display increased levels of KEAP 1 and reduced expression of Nrf2 [41]. 

Previously, we showed that α-LA inhibits the expression of IL-8 in *H. pylori*-infected gastric epithelial AGS cells by suppressing inflammatory signaling pathways including those of mitogen-activated protein kinases, JAK/STAT and NF-kB [7]. We have also reported that α-LA reduces ROS levels in *H. pylori*-infected AGS cells by suppressing the activation of NADPH oxidase [8]. The results of the present study define the role that the Nrf2/HO-1 plays in α-LA-induced reduction in ROS and IL-8 levels in *H. pylori*-infected AGS cells. We also found that the suppression of Nrf2-KEAP 1 complex formation is the mechanism by which α-LA activates the Nrf2/HO-1 pathway in *H. pylori*-infected AGS cells. 

Trigonelline is an alkaloid and occurs in many plants including coffee [42]. It is a specific small-molecule Nrf2 inhibitor and inhibits cell migration through downregulation of Nrf2–dependent antioxidant enzymes’ activity [43]. Inhibition of the Nrf2 transcription factor was observed by trigonelline which has the potential to be used in combination therapy of highly-resistant tumors such as pancreatic cancer [44]. 

ZnPP is a metabolite formed in the process of heme biosynthesis. The lack of iron leads to increased ZnPP formation in the blood. High levels of ZnPP in the blood play a role in the inhibition of HO-1, which is the rate-limiting enzyme in the heme degradation pathway [45]. Since inhibition of HO-1 by ZnPP suppresses tumor cell growth [46], ZnPP has been suggested as a useful agent for antitumor therapy [47]. Therefore, ZnPP has been used as a potent competitive inhibitor of HO-1 in experimental studies.

Regarding *H. pylori* strain, Yamaoka et al. [22] examined expression patterns of IL-8 using real-time PCR analysis and ELISA in gastric biopsy specimens of 192 patients. *CagA* gene was determined using PCR. They reported that *H pylori* infection was associated with increased rate of mRNA expression of IL-8 and with increased mucosal level of IL-8. IL-8 levels were correlated with the density of *H pylori* in both the antrum and corpus. *CagA* gene positive *H pylori* infection was related to increased expression of IL-8 at mRNA and protein levels. NCTC 11637, a *cagA*-positive strain, was the first *Helicobacter* cultured and has been distributed through national reference culture collections to researchers throughout the world [48]. Therefore, *cag A* positive *H. pylori* strain, NCTC 11637, was used on the effect of α-LA on IL-8 expression in the present study. 

From the aspect of gastric cells, the human gastric adenocarcinoma AGS or MKN-28 cells, gastric carcinoma KATO III cells, and rat gastric mucosal cells (RGM-1 which is non-adenocarcinoma cells) have been used for studies on gastric diseases caused by *H. pylori* infection. However, the majority of studies on cytokine responses to *H.* *pylori* infection in vitro have used AGS and MK-28 cells. The studies, using AGS or MKN-28 cells, showed different cellular responses to *H. pylori*. While *H. pylori*-infected AGS cells show rapid cell elongation within only a few h, MKN-28 cells respond with a clear delay [49]. In addition, AGS secretes significantly more IL-8 than MKN-28 [50]. Therefore, many studies that investigate a relationship between *H. pylori* and inflammatory cytokine response have used AGS cells.

The concentration of α-LA used in the present study is 5 μM. Several studies demonstrated that the administration of α-LA at a dose of 600 mg improved insulin sensitivity in patients with type 2 diabetes mellitus and cognitive functions in patients with Alzheimer's Disease [3,4,5,6]. Ikuta et al. [51] showed that the plasma concentration of α-LA after a single oral administration of 600 mg of α-LA in healthy humans was 1.68 µg/mL (approximately 8.1 μM). Therefore, 5 μM α-LA used in this study is physiologically relevant. In addition, the effect of pretreatment with α-LA on *H. pylori*-induced IL8 expression in gastric epithelial cells was determined in the present study. Therefore, the results are relevant to prevention against *H. pylori* infection.

Our findings support the hypothesis that consumption of α-LA-rich foods might prevent the development of *H. pylori*-induced gastric inflammation by decreasing ROS-mediated IL-8 expression through the Nrf2/HO-1 pathway in gastric epithelial cells.

## 5. Conclusions

α-LA activates the Nrf2/HO-1 pathway by decreasing the interaction between Nrf2 and KEAP 1 and thus, reducing ROS levels and IL-8 expression in *H. pylori*-infected AGS cells. Consumption of α-LA-rich foods may prevent the development of *H. pylori*-associated gastric inflammation.
